# Supplementary figures and images for: Nanoresolution real-time 3D orbital tracking for studying mitochondrial trafficking in vertebrate axons in vivo
Source: eLife. 2019 Jun 10;8:e46059. doi: 10.7554/eLife.46059 (PMC6579510; doi:10.7554/eLife.46059)

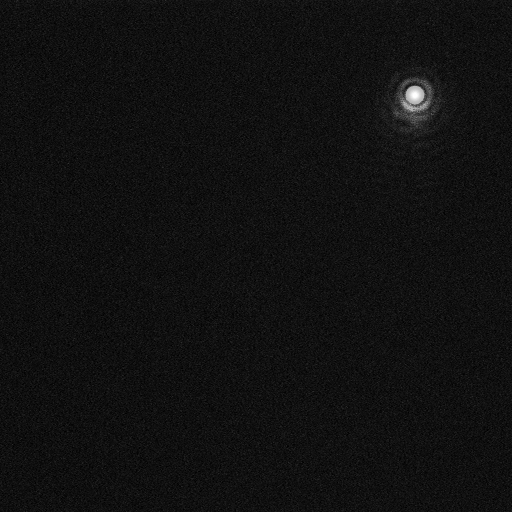

Supplement: Figure 3—source data 1. [file elife-46059-fig3-data1.zip › Figure 3 - Source Data and Code/Panel C/Repetitive 3 - 2015.07.09/Mapping Calibration.tif]
